# Next‐generation sequencing for *BCR‐ABL1* kinase domain mutations in adult patients with Philadelphia chromosome‐positive acute lymphoblastic leukemia: A position paper

**DOI:** 10.1002/cam4.2946

**Published:** 2020-03-10

**Authors:** Simona Soverini, Francesco Albano, Renato Bassan, Francesco Fabbiano, Felicetto Ferrara, Robin Foà, Attilio Olivieri, Alessandro Rambaldi, Giuseppe Rossi, Simona Sica, Giorgina Specchia, Adriano Venditti, Giovanni Barosi, Fabrizio Pane

**Affiliations:** ^1^ Institute of Hematology “Lorenzo e Ariosto Seràgnoli” Department of Experimental, Diagnostic and Specialty Medicine University of Bologna Bologna Italy; ^2^ Department of Emergency and Organ Transplantation (D.E.T.O.) Hematology Section University of Bari Bari Italy; ^3^ Ospedale dell'Angelo UOC Ematologia Mestre‐Venezia Italy; ^4^ UOC Ospedali Riuniti Villa Sofia‐Cervello Palermo Italy; ^5^ Division of Hematology Cardarelli Hospital Naples Italy; ^6^ Division of Hematology University "Sapienza" Rome Italy; ^7^ Department of Hematology Università Politecnica delle Marche Ancona Italy; ^8^ Department of Oncology and Hemato‐Oncology University of Milan and Azienda Socio‐Sanitaria Territoriale (ASST) Ospedale Papa Giovanni XXIII Bergamo Italy; ^9^ Dipartimento di Oncologia Clinica A.O. Spedali Civili Brescia Italy; ^10^ Fondazione Policlinico Universitario A. Gemelli Rome Italy; ^11^ Università Cattolica del Sacro Cuore Rome Italy; ^12^ Dipartimento di Biomedicina e Prevenzione Universitá Tor Vergata Rome Italy; ^13^ IRCCS Policlinico S. Matteo Foundation Pavia Italy; ^14^ U.O.C. Ematologia e Trapianti di Midollo Azienda Ospedaliera Universitaria Federico II di Napoli Naples Italy

**Keywords:** acute lymphoblastic leukemia, *BCR‐ABL1* tyrosine kinase, consensus development, next‐generation sequencing, Philadelphia chromosome, point mutation, Sanger sequencing

## Abstract

Emergence of clones carrying point mutations in the *BCR‐ABL1* kinase domain (KD) is a common mechanism of resistance to tyrosine kinase inhibitor (TKI)‐based therapies in Philadelphia chromosome‐positive (Ph+) acute lymphoblastic leukemia (ALL). Sanger sequencing (SS) is the most frequently used method for diagnostic *BCR‐ABL1* KD mutation screening, but it has some limitations—it is poorly sensitive and cannot robustly identify compound mutations. Next‐generation sequencing (NGS) may overcome these problems. NSG is increasingly available and has the potential to become the method of choice for diagnostic *BCR‐ABL1* KD mutation screening. A group discussion within an ad hoc constituted Panel of Experts has produced a series of consensus‐based statements on the potential value of NGS testing before and during first‐line TKI‐based treatment, in relapsed/refractory cases, before and after allo‐stem cell transplantation, and on how NGS results may impact on therapeutic decisions. A set of minimal technical and methodological requirements for the analysis and the reporting of results has also been defined. The proposals herein reported may be used to guide the practical use of NGS for *BCR‐ABL1* KD mutation testing in Ph+ ALL.

## Graphical abstract

This consensus paper presents the results of an initiative by an expert panel to define a set of indications for the practical use of next‐generation sequencing for *BCR‐ABL1* kinase domain mutation screening in Philadelphia‐positive acute lymphoblastic leukemia patients receiving tyrosine kinase inhibitor‐based therapies. Minimal technical and methodological requirements for the analysis and the reporting of results have also been proposed.

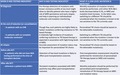

## 1 INTRODUCTION

The introduction of tyrosine kinase inhibitors (TKIs) for the treatment of adult Philadelphia‐positive (Ph+) acute lymphoblastic leukemia (ALL) has dramatically increased complete hematological response (CHR) rates from 60%‐70% to 95%‐100% and extended 5‐year overall survival (OS) from 20% up to 50%.^1^, ^2^ A number of studies have tested the combination of TKIs with more or less intensive chemotherapy regimens and schedules and even the use of chemotherapy‐free approaches, first in elderly and unfit patients and more recently in unselected adult patients.^3^, ^4^, ^5^ In all studies, when relapses occurred, point mutations in the *BCR‐ABL1* kinase domain (KD) were detected in 50%‐80% of patients.^5^ These mutations displayed high IC_50_ values in biochemical and cellular assays^6^, ^7^, ^8^ implying that they have a direct causal role in TKI resistance.^9^, ^10^, ^11^, ^12^, ^13^ The most frequent imatinib‐resistant mutation in Ph+ ALL patients is the T315I,^14^, ^15^ against whom second‐generation TKIs (2GTKIs), but not ponatinib or monoclonal antibodies, are ineffective.^16^, ^17^, ^18^, ^19^ Patients positive for mutations exhibit a particularly high degree of genetic instability, which may foster the acquisition of additional mutations in the same (“compound” mutations) or in different (“polyclonal” mutations) Ph+ subpopulations, generating complex mutation landscapes.^20^, ^21^ Compound mutations are particularly challenging to address, since IC_50_ data suggest that they are highly resistant to imatinib and all second‐generation TKIs.^22^

There is increasing evidence that a rational use of minimal residual disease (MRD) monitoring and *BCR‐ABL1* KD mutation screening may play an important role in treatment optimization.^23^, ^24^ However, in contrast to chronic myeloid leukemia (CML), recommendations on when and how to perform *BCR‐ABL1* KD mutation screening in Ph+ ALL have never been produced. Mutation testing is commonly performed by bidirectional Sanger sequencing (SS) of the entire *BCR‐ABL1* KD amplified by polymerase chain reaction.^25^ However, SS fails to reveal the presence of mutations when they are present in less than 10%‐20% of *BCR‐ABL1* transcripts.^25^ Thus, a number of alternative methodologies, including next‐generation sequencing (NGS), have been proposed to facilitate earlier detection of mutant, potentially resistant *BCR‐ABL1*‐positive cells.^26^, ^27^, ^28^, ^29^, ^30^, ^31^, ^32^, ^33^, ^34^ Routine NGS testing is being implemented in a greater and greater number of diagnostic laboratories. It is well documented that NGS is markedly superior to SS in terms of sensitivity, and can additionally allow straightforward identification of the most frequent compound mutations.^26^, ^27^, ^28^, ^29^, ^30^, ^31^, ^32^, ^33^, ^34^ However, very few data are available on the clinical impact of *BCR‐ABL1* KD mutation testing by NGS in patients with Ph+ ALL, and this does not allow the formulation of evidence‐based recommendations. The aim of this project was thus to generate consensus‐based indications for the possible clinical use of NGS in Ph+ ALL.

## 2 METHODS

A Panel of Experts was appointed on the basis of their nationally and internationally recognized expertise in the treatment and molecular monitoring of ALL. An initial meeting was held in June 2018, during which the outline of the project was defined and the topics of the expert discussion were decided. A series of key questions were identified and addressed through questionnaires. Each panelist drafted statements related to one or a few questions, while the remaining panelists scored their agreement with those statements or provided suggestions for modifications. Subsequently, the Panel convened for a consensus conference in Milan in November 2018, where final proposals were defined using the nominal group technique.^35^ Briefly, Panel members were first asked to comment in a round‐robin fashion on their disagreements on each issue, and then to vote for a final statement.

## 3 RESULTS

### 3.1 NGS testing before and during frontline treatment

When using highly sensitive methods of detection, *BCR‐ABL1* KD mutations have been identified in de novo Ph+ ALL.^10^, ^12^, ^13^, ^29^, ^36^, ^37^, ^38^, ^39^ Some patients, in particular, may already be harboring the pan‐resistant T315I mutation at the time of diagnosis.^12^, ^36^, ^39^

Using denaturing high‐performance liquid chromatography (D‐HPLC), Pfeifer et al^12^ documented the detection of a KD mutation in 41% of Ph+ ALL patients prior to induction therapy. The frequency of the mutant allele was always low, ranging from 0.1% to 2% (median, 0.5%). Patients were enrolled in a prospective, randomized clinical trial of the German Multicenter Study Group for Adult ALL, exploring frontline imatinib‐based therapy in elderly patients, and were randomly assigned to receive a 4‐week induction with either imatinib or chemotherapy followed by extended therapy with imatinib plus chemotherapy. Pretherapy mutations were not associated with a poorer hematologic or molecular remission rate or shorter remission duration. Despite the high complete response rate, however, all but two patients who were initially found to have a KD mutation, and did not die in complete response, subsequently relapsed. At relapse, the dominant clone showed an identical mutation in 90% of cases, suggesting selection of the pre‐therapy mutation. Only one patient relapsed with a different mutation.

Rousselot et al^37^ retrospectively performed allele‐specific oligonucleotide polymerase chain reaction (ASO‐PCR) on baseline samples available from 43 cases enrolled in a clinical trial of low‐intensity chemotherapy plus dasatinib in Ph+ ALL patients older than 55 years. The analysis showed that in 10/43 samples a T315I mutation was already present at very low levels at diagnosis. Eight of these 10 patients relapsed, all with the T315I mutation; of the 2 who did not relapse, one patient was transplanted and was still in complete response 54 months after allogeneic hematopoietic stem cell transplant (allo‐SCT) and the other died in CHR at 9.6 months from lung adenocarcinoma. Additional anecdotal cases in which the mutation detected at the time of relapse could be traced back to the pretherapy or diagnosis sample can be found in the literature.^10^, ^13^, ^29^, ^39^

Real‐time PCR‐based assessment of residual *BCR‐ABL1* transcript levels in Ph+ ALL patients is universally accepted as a strong prognostic factor and is performed at regular intervals during treatment.^40^ Persistence of MRD positivity and/or MRD increase may predict the emergence of TKI‐resistant *BCR‐ABL1* KD mutations.^29^ NGS‐based monitoring of the dynamics of emerging *BCR‐ABL1* mutations in parallel to MRD assessment may thus provide meaningful information on the subclonal sensitivity of Ph+ leukemic cells to the current therapy. As a matter of fact, a strikingly rapid expansion of the mutated clones as early as after 4 weeks since TKI initiation was observed,^29^, ^36^ suggesting that, at least in a proportion of patients, the mechanism of therapy resistance may be due to the selection of preexisting mutated clones rather than or in addition to the induction of new mutations. Thus, only serial and frequent monitoring can intercept the very initial phases of clonal selection.

#### 3.1.1 Consensus statements

*BCR‐ABL1* KD mutation testing by NGS is indicated in patients with Ph+ ALL before frontline treatment. The Panel argued that the detection of *BCR‐ABL1* KD mutations that might lead to the emergence of TKI‐resistant clones could allow the identification of patients at a higher risk of MRD persistence and early relapse, and help in planning an individualized molecular and mutation monitoring.

In patients with pretherapy low‐level mutations known to confer resistance to one or more TKIs, monthly retesting should be performed to monitor mutation kinetics, until residual *BCR‐ABL1* transcript levels decrease below 0.1%, or until the increase in mutation and *BCR‐ABL1* transcript levels leads to therapeutic intervention.

### 3.2 NGS testing in refractory and relapsing patients

Relapses after therapy may be associated with the emergence or persistence of clones harboring mutations that confer resistance to the TKI incorporated in the treatment regimen. Since for each TKI there is a well‐defined spectrum of sensitive and resistant mutations, an accurate assessment of *BCR‐ABL1* KD mutation status is important for treatment decision‐making.

Using NGS, Soverini et al^28^ analyzed longitudinally 106 samples from 33 patients with CML or Ph+ ALL who had received multiple lines of TKI treatment and who had experienced sequential relapses accompanied by the emergence of one or more mutations. They found that SS had misclassified or underestimated the *BCR‐ABL1* KD mutational status in 55% of samples. Indeed, low‐level mutations were detected not only in SS‐negative patients, but also in addition to the dominant clone(s) detected by SS. Compound mutations were frequent in advanced phase CML and in Ph+ ALL, and could easily be recognized by NGS in the majority of cases. In another study,^29^ Soverini et al analyzed by NGS 106 samples from 34 patients with Ph+ ALL who relapsed after first (n = 10 pts) or second/subsequent line (n = 24) of TKI treatment in an attempt to “backtrack” mutation emergence. NGS detected resistance‐driver mutations earlier than SS in 41% of patients. All samples positive for low‐level mutations had persistently high or rising levels of MRD. NGS of the *BCR‐ABL1* KD proved capable of anticipating an impending recurrence with a median of 5 weeks (range, 4‐14 weeks) compared to the classical SS‐based analysis.

#### 3.2.1 Consensus statements

*BCR‐ABL1* KD mutation testing by NGS is indicated in patients who do not reach a CHR after induction therapy.

*BCR‐ABL1* KD mutation testing by NGS is also indicated in patients who do not reach a complete molecular response after induction therapy. In the latter, however, the BCR‐ABL1 transcript level should be >0.1% to ensure the feasibility of NGS library preparation. Given the variability of the induction protocols, at least 4 weeks of induction therapy should be completed before NGS testing.

*BCR‐ABL1* KD mutation testing by NGS testing is indicated in patients with a relapsing disease before receiving salvage therapy. The Panel argued that the information provided by NGS testing in refractory and relapsing patients may allow the personalization of an optimal TKI treatment choice.

### 3.3 NGS testing before and after allogeneic hematopoietic stem cell transplant

Allogeneic hematopoietic stem cell transplant (allo‐SCT) is still considered the best option in attempting to pursue long‐term disease control in Ph+ ALL.^23^ According to the European Group for Blood and Marrow Transplantation, patients with undetectable MRD after allo‐SCT may be treated prophylactically or, alternatively, may be monitored and receive a TKI only if and when they convert to MRD‐positivity, whereas patients with detectable MRD after allo‐SCT should be started on TKI treatment as soon as possible.^41^

Several retrospective, comparative analyses have been performed with the aim of evaluating the impact of the use of TKIs after allo‐SCT on outcome. The largest analysis, restricted to patients treated with allo‐SCT in first complete remission, showed that imatinib or dasatinib for primary prophylaxis against relapse resulted in an improvement of OS and leukemia‐free survival along with a lower incidence of grade 2‐4 acute graft versus host disease.^42^ Although the study had some important limitations associated with its retrospective nature, the results provide a strong rationale for the use of TKIs as maintenance after allo‐SCT for patients with Ph+ ALL in first remission.

Unfortunately, TKI resistance mechanisms in the transplantation setting have not been extensively investigated; indeed, there are very few data about the mutational status of MRD‐positive patients both before and after allo‐SCT, as well as about the patterns of clonal evolution of preexisting mutations. Egan et al^43^ have shown that Ph+ leukemia patients harboring *BCR‐ABL1* KD mutations before allo‐SCT predominantly relapse with the same mutation. Therefore, empiric selection of any one TKI in the posttransplant management will almost certainly result in predictable treatment failures in a subset of patients with preexisting mutations. In addition to considerations of cost and toxicity, the pretransplant mutation status of patients with detectable residual disease should thus be considered when choosing the TKI for posttransplant prophylaxis.

#### 3.3.1 Consensus statements

The Panel agreed that knowledge of the *BCR‐ABL1* KD mutation status prior to an allo‐SCT could provide useful information about the risk of recurrence of the disease after transplant and about the posttransplant TKI to be utilized. Thus, patients who did not have NGS testing done at the time of transplant decision should have it carried out prior to the transplant (if the *BCR‐ABL1* transcript level is greater than 0.1%).

After transplant, *BCR‐ABL1* KD mutation screening by NGS should be performed whenever a patient tests MRD positive, with *BCR‐ABL1* levels greater than 0.1%. If a low‐level mutation is detected, mutation kinetics should be monitored at monthly intervals, and the TKI to which the mutation is known to confer resistance should be avoided.

### 3.4 Impact of NGS on therapeutic decisions

In Ph+ ALL patients who fail imatinib treatment, *BCR‐ABL1* mutation profiling may be useful before changing therapy since the detection of specific mutations may influence the choice of the second‐generation TKI.^23^, ^24^ This is particularly relevant now that the approval of ponatinib offers significant chances to rescue patients with the T315I or with multiple mutations predicted to result in insensitivity to both dasatinib and nilotinib.^44^ Detailed lists of imatinib‐, dasatinib‐, nilotinib‐, and bosutinib‐resistant mutations are available to support clinicians in this decision‐making.^5^ However, even though scientifically sound, the hypothesis that mutation‐guided TKI selection would result in better outcomes than an empirically based TKI selection has not yet been tested and validated in prospective clinical trials.

The setting in which the greater sensitivity of NGS could be most useful is when a Ph+ ALL patient with a nonoptimal response to imatinib has to be shifted to second‐line therapy, and in the transplant setting. Typically, patients candidate for the use of second‐generation TKIs in the pretransplant or posttransplant setting are represented by those who have experienced resistance to pretransplant treatment with imatinib. In fact, in the posttransplant setting, early prophylactic or MRD‐triggered imatinib was not always effective in reducing the risk of clinical or molecular relapse.^45^ Nilotinib has been tested after SCT in a single prospective trial,^46^ whereas dasatinib only in small retrospective cohorts.^47^, ^48^, ^49^, ^50^ The use of third‐generation TKIs, such as ponatinib, as maintenance after allo‐SCT has not been reported so far. Thus, there are no accepted criteria for choosing an alternative TKI after imatinib, and this decision should be based both on the safety profile and on the predictable efficacy.

#### 3.4.1 Consensus statements

In general, the interpretation of NGS testing results and the therapeutic decisions should involve both biologists/biotechnologists and physicians expert in Ph+ ALL monitoring and treatment, respectively.

Since there is no established evidence of a real clinical advantage of a personalized therapy tailored according to the detection of low‐level mutations, at the present time, any therapeutic decision based on the results of NGS testing may only derive from inductive reasoning.

The majority of Panel members agreed that the NGS results obtained prior to front‐line therapy should restrain from modifying the approved conventional treatment, but should rather trigger monthly evaluation of mutation kinetics (in the presence of quantifiable disease).

On the contrary, the identification of a low‐level *BCR‐ABL1* KD mutation by NGS in patients who are MRD‐positive after TKI‐based induction or consolidation should prompt to consider switching TKI when the mutation is known to be not sensitive to the TKI currently used by the patient. In this case, the detection of mutations by NGS should encourage an individualized therapy or a therapeutic switch that depends on the type and level of mutation(s) and on the toxicity profile of the various options.

The Panel claimed that *BCR‐ABL1* KD mutation testing by NGS has an important role in patients who are candidate for the posttransplant use of TKIs in order to reduce the risk of relapse. In the case of persistence or reappearance of MRD positivity after allo‐SCT, the choice of an alternative TKI should be based on the results of NGS analysis.

### 3.5 Strength of indications of NGS testing and logistic issues

The Panel addressed the matter of how mandatory the indications for the use of NGS testing for *BCR‐ABL1* KD mutation issued by this document should be for clinical centers that do not have access to the NGS technology. The Panel agreed that NGS testing should be encouraged, in the proper indications of use, in all the clinical centers caring for patients with Ph+ ALL. However, the Panel also acknowledged that there is currently no evidence demonstrating that the lack of NGS testing hampers the possibility to properly manage Ph+ ALL patients, thus failure to use NGS because of the lack of access to it, at present, should not be regarded as an inappropriate clinical management.

The Panel also advocated that NGS testing should be performed in a restricted number of highly qualified laboratories within regional or national networks, already engaged in MRD monitoring of Ph+ ALL patients. Important aims of these networks should be: centralization of the analysis in a few highly specialized laboratories; harmonization of techniques among the network laboratories; central coordination for continuous technology development and also through worldwide collaborations with other reference laboratories; development of an inter‐lab quality control system for sensitivity, accuracy, and reproducibility of the results; organization of periodical quality control rounds.

### 3.6 Performance characteristics of NGS testing in Ph+ ALL

**Figure 1:**
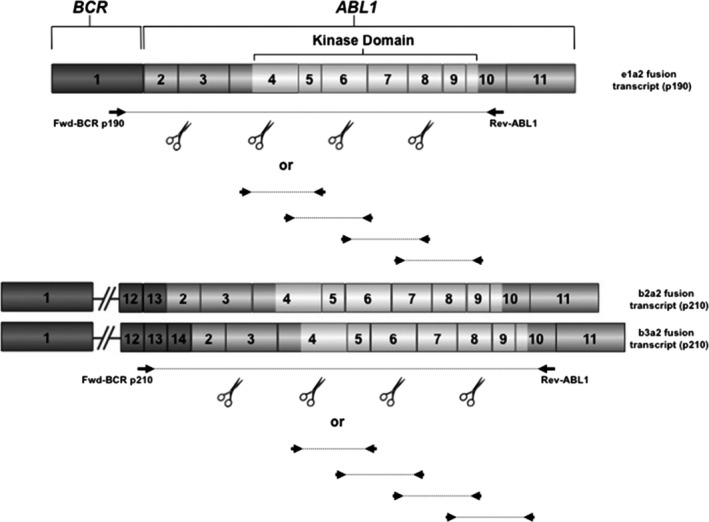
Schematic representation of assay design for next‐generation sequencing‐based *BCR‐ABL1* kinase domain (KD) mutation screening. After RNA extraction from bone marrow mononuclear cells and reverse transcription, selective amplification of the *ABL1* KD from the fusion *BCR‐ABL1* allele may be accomplished by using two alternative forward primers, either on *BCR* exon 1 (for the e1a2 fusion; p190) or on exon 12/13 (for the b2a2 and b3a2 fusions; p210) and a common reverse primer on *ABL1* exon 10. The resulting amplicon may be either used as a template for a nested polymerase chain reaction or may be enzymatically fragmented, provided that the resulting amplicons, or fragments, are not shorter than 300‐400 bp, to maximize the likelihood to detect compound mutations. The kinase domain encompasses amino acids 235‐498

Although in Ph+ ALL P‐loop mutations and T315I are the most frequent,^15^, ^36^ imatinib‐resistant mutations have been observed all over the KD. For this reason, the European LeukemiaNet recommendations on *BCR‐ABL1* mutation testing in CML underlined the importance of using sequencing approaches rather than mutation‐specific assays, enabling screening for a limited number of mutations only. No commercial assay is, as yet, available.^51^ A series of studies have described the setup of home brew protocols, all using RNA as a starting material and selectively amplifying the KD of translocated *ABL1*, implemented on various NGS platforms.^26^, ^27^, ^28^, ^29^, ^30^, ^31^, ^32^, ^33^, ^34^ Such studies have suggested that *BCR‐ABL1* KD mutation screening by NGS is feasible in expert laboratories, and that NGS results are accurate and reproducible down to variant frequencies of 1%^26^, ^27^, ^28^, ^29^, ^30^, ^31^, ^32^, ^33^—although a recent multicenter study has highlighted that 3% is a more robust threshold.^34^ A schematic representation of a proposed assay design for NGS‐based *BCR‐ABL1* KD mutation screening is presented in Figure 1.

#### 3.6.1 Consensus statements

Bone marrow (BM) should be the preferred specimen for *BCR‐ABL1* KD mutation screening in Ph+ ALL. At least 5 mL of BM should be obtained using ethylenediaminetetraacetic acid or sodium citrate as an anticoagulant; heparin must be avoided. RNA from mononuclear cells is the recommended template. Selective amplification of the *ABL1* KD of the fusion *BCR‐ABL1* allele is recommended. This may be accomplished using either of two forward primers, on *BCR* exon 1 (specific for the e1a2 fusion, encoding p190^BCR‐ABL1^) or on *BCR* exons 12/13 (specific for the b2a2 and b3a2 fusions, encoding p210^BCR‐ABL1^) and a reverse primer on *ABL1* exon 10. The resulting amplicon may be either used as a template for a nested PCR or may be fragmented, provided that the final amplicons or fragments are not shorter than 300‐400 bp, to maximize the likelihood to detect compound mutations. Accordingly, sequencing chemistries/cycles producing reads shorter than 400 bp are not recommended. For the above reasons, commercial gene panels incorporating *ABL1* but using genomic DNA as input material and sequencing short fragments are not recommended.

The minimal mRNA region to be screened for mutations is that encoding amino acids 235 through 498 of the *ABL1* 1a protein isoform, known to correspond to the KD (reference Genbank sequence: NM_005157.5). No mutations should be reported outside this region.

The recommended minimum depth of coverage is 1000×. Unless commercial kits become available, each individual laboratory will be responsible for the optimization of assay conditions and for the evaluation of accuracy, precision and analytical sensitivity. Given the inherent differences between NGS platforms, chemistries, and bioinformatics tools, specific recommendations on ranges and thresholds cannot be provided. To ensure optimal analytical performance, each laboratory will have to define means and criteria for quality control and to engage in regular proficiency testing programs. The Panel suggests that mutations with a variant allele frequency below 3% should not be reported. Any variant >3% should be reported irrespective of the availability of experimental or clinical information regarding its sensitivity profile. Laboratory reports should include the following minimal set of information:

- whether the sample is evaluable or not and why (insufficient RNA quality/quantity; unsuccessful amplification of the *ABL1* KD of the *BCR‐ABL1* transcript);
- for each variant detected, listed as nucleotide and amino acid substitution, the relative frequency and a clear indication of whether it has a known or unknown resistance profile. The TKI(s) to whom the variant is known to be insensitive should be clearly indicated.

The turnaround time for reporting results should be 2 weeks or shorter.

## 4 DISCUSSION

Many laboratories are currently in the process of introducing NGS into their routine diagnostic procedures since it has proven a robust, reproducible and, in the case of gene panels, cost‐effective alternative to SS. Here, Ph+ ALL experts analyzed the body of data on the use of NGS for *BCR‐ABL1* KD mutation testing in Ph+ ALL and judged whether it was sufficiently robust to provide recommendations. So far, there has been no randomized clinical trial formally assessing the efficacy of a proactive change of therapy based on the detection of low‐level mutations by NGS, and considering the low incidence of Ph+ ALL cases and the heterogeneity of TKI and chemotherapy combination regimens used by different cooperative study groups, such a trial will hardly be feasible. This forced the expert Panel to use the method of consensus to shape the statements herein presented.

The Panel assessed the potential value of NGS testing in three distinct settings: before and during first‐line TKI‐based treatment; in relapsed/refractory cases; before and after allo‐SCT. The first was the most debated setting. The Panel discussion highlighted the lack of prospective data and the paucity of retrospective data—all obtained with other technologies (ASO‐PCR, D‐HPLC, ligation‐dependent PCR) characterized by variable levels of sensitivity, accuracy, and reproducibility. In the great majority of cases, the same TKI‐resistant mutation detected at low levels at diagnosis coincided with that harbored by the dominant clone detected at relapse, although some exceptions (patients not relapsing or relapsing with a different mutation) have been reported.^12^, ^36^, ^37^ Based on observations made in CML, where more data are available, a mutant clone present at low levels can be expected to undergo Darwinian selection if the TKI is not active against the mutation. However, if and how the associated chemotherapy may modify the selective pressure of TKIs, and whether more or less intensive chemotherapy regimens may exert different roles, remains to be assessed in Ph+ ALL. Based on the above considerations, the majority of the Panel members agreed that NGS at diagnosis is indicated, but that NGS results should be restrained from modifying the approved conventional therapeutic protocol. NGS should rather be used to identify patients at a higher risk of early relapse, defined as those carrying at baseline a TKI‐resistant low‐level mutation by NGS. These patients should undergo an individualized molecular and mutation monitoring, with monthly NGS testing recommended (in the presence of detectable disease) to follow mutation kinetics in order to consider a timely mutation‐driven preemptive therapy if MRD remains persistently high or tends to increase over time. As a matter of fact, it has been reported that resistance‐driven mutations expand very rapidly.^29^ Although no data is currently available that demonstrate the benefits of tailoring front‐line therapy of Ph+ ALL patients based on NGS‐detectable mutations at diagnosis, in the era of personalized medicine such an approach indisputably represents an attractive opportunity. Thus, prospective trials should be designed in the near future to properly address this important point.

The Panel, that also included experts in molecular biology and NGS testing, additionally engaged in the definition of a set of minimal technical and methodological requirements for analysis and result reporting. They also underlined that NGS should be implemented only in a limited number of highly qualified laboratories already involved in MRD monitoring of Ph+ ALL patients, so that *BCR‐ABL1* KD mutation screening may be activated in a timely manner on the same RNA or cDNA samples used for MRD analysis whenever necessary. The centralization of testing will also be required for cost‐effectiveness. Several regional or national networks of expert laboratories are already in place, which in turn are involved in worldwide cooperative efforts (eg, ESLHO—European Scientific foundation for Laboratory HematoOncology). It would be advisable for such networks to take the lead in future standardization and organization of periodical quality control rounds. Harmonization of protocols and coordinated definition of standard requirements for sensitivity, accuracy, and reproducibility of the results are particularly critical issues. At present, unfortunately, no commercial kit for NGS‐based *BCR‐ABL1* KD mutation testing yet exists. A common NGS kit to be standardized across centers is eagerly awaited, since that would increase the number of centers that can perform in house analyses.

From a technical point of view, NGS‐based *BCR‐ABL1* KD mutation screening can be performed on the same RNA or cDNA used for MRD testing. Indeed, real‐time PCR should always be performed first, and MRD results should guide the decision of whether to proceed to NGS testing. It has been reported that MRD persistence in Ph+ ALL patients may hide emerging clinically actionable mutations that NGS may timely identify, thus enabling treatment optimization aimed to prevent hematologic relapse.^29^ For this reason, the Panel stated that NGS is indicated not only in patients who fail to achieve or who lose CHR, but also in those who test MRD positive at the end of induction or consolidation therapy. Similarly, the Panel recognized that NGS might provide valuable information in patients who display MRD positivity after transplant. Knowledge of *BCR‐ABL1* transcript levels is also important to assess the feasibility of NGS testing. In CML samples, it has been reported that at values below 0.1% library preparation is not always successful, and even when it is successful, repeatability is reduced, and false negative results are not infrequent.^52^

In conclusion, we here report the first attempt at defining indications for the use of NGS for *BCR‐ABL1* KD mutation screening in Ph+ ALL. Table 1 presents an overview of our consensus‐based indications. This initiative was fostered by the awareness that enhancing the capability of detecting emerging mutations and providing the most accurate picture of the mutation status may bring important advantages in defined patient settings. At the same time, the Panel of Experts also felt that, in order to avoid inappropriate use of non‐standardized testing, a minimal set of indications had to be defined as to when and how to perform NGS testing. A similar effort has recently been accomplished for *BCR‐ABL1* KD mutation testing in CML.^53^

**Table 1:** Summary of the indications of use of next‐generation sequencing (NGS) in Philadelphia‐positive acute lymphoblastic leukemia (ALL)

When is NGS testing indicated?	Why is NGS testing indicated?	What if a low‐level mutation is detected?
At diagnosis	Pretherapy detection of mutations with known resistance profile at low level might allow to identify patients who have a higher risk of MRD persistence and early relapse, and help in planning an individualized molecular and mutation monitoring	Monthly evaluation of mutation kinetics should be performed, until either *BCR‐ABL1* levels decrease below 0.1%, or MRD and mutation level increase In the latter case, switching to a different TKI should be considered when the mutation is known to be not sensitive to the TKI currently used
At the end of induction (or consolidation)		
In patients with no CHR	Though few, such patients are highly likely to harbor mutations conferring resistance to TKI‐based therapy	Personalized TKI choice should be based on the detectable mutation(s)
In patients with no complete molecular remission^a^	A relatively high incidence of mutations conferring resistance to TKI‐based therapy is expected in association with high/rising levels of MRD	Switching to a different TKI should be considered when the mutation is known to be not sensitive to the TKI currently used
At relapse	Accurate assessment of mutation status may be important for personalized TKI choice	Personalized TKI choice should be based on the detectable mutation(s)
Before allo‐SCT		
In patients who did not have NGS testing performed at the time of transplant decision^a^	Detection of low‐level mutations is likely to affect posttransplantation outcome	Posttransplantation reassessment should be performed for reinstitution of personalized TKI therapy based on MRD and mutation status
After allo‐SCT		
Whenever a patient tests MRD+^a^	Persistent mutations associated with MRD positivity may affect posttransplantation outcome	Monthly evaluation should be performed for personalized posttransplantation reinstitution of TKI therapy based on MRD and mutation status

Abbreviations: allo‐SCT, allogeneic hematopoietic stem cell transplant; CHR, complete hematological response; MRD, minimal residual disease; TKI, tyrosine kinase inhibitor.

a *BCR‐ABL1* transcript level should be >0.1% to ensure the feasibility of NGS library preparation.

Although a consensus on a series of position statements was reached, the literature review and the Panel discussion highlighted the strong need for prospective studies aimed at systematically applying NGS for *BCR‐ABL1* KD mutation monitoring of Ph+ ALL patients in the framework of TKI‐based clinical trials. Such studies, that would greatly benefit from the international cooperation of different study groups, would help to establish the role of NGS at diagnosis and to find the best timing and integration of MRD and mutation testing into the clinical decision algorithms.

## CONFLICT OF INTEREST

Simona Soverini has received honoraria from Incyte Biosciences, Novartis, Bristol‐Myers Squibb. Francesco Albano has received honoraria for advisory board from Incyte. Renato Bassan has received honoraria advisory board from Amgen, Pfizer, Shire, Servier, Jazz, Incyte. Francesco Fabbiano has received honoraria for advisory board from Incyte. Robin Foà has received honoraria for consultancies/advisory board from Janssen, Novartis, Amgen, Abbvie, Roche, Incyte, Shire, Pfizer. Attilio Olivieri has received honoraria for advisory board from Incyte. Alessandro Rambaldi has received honoraria for travel, accommodation expenses and fees for consultancies and participation to meetings, boards and symposia sponsored by Amgen, Pfizer, Incyte, Italfarmaco, Astellas, Celgene, Novartis, Roche, Jazz. Giuseppe Rossi has received honoraria from Novartis, BMS, Incyte and participated to advisory boards from Amgen, Pfizer. Simona Sica has received honoraria for advisory board from Incyte, Sanofi, Alexion. Adriano Venditti has received personal fees from Pfizer, Novartis, Astellas, Jazz Pharmaceuticals, Amgen, Incyte and Daiichi‐Sankyo outside the submitted work. Fabrizio Pane has received honoraria for advisory board from Amgen, Pfizer. The remaining authors have no conflicts of interest to disclose.

## AUTHORS' CONTRIBUTIONS

All authors contributed to conception and design of the work, analysis, and interpretation of the literature and manuscript drafting and gave final approval for submission.

## Data Availability

Data sharing is not applicable to this article as no new data were created or analyzed in this study.
